# Continuum of care for persons with common mental health disorders in Nunavik: a descriptive study

**DOI:** 10.3402/ijch.v74.27186

**Published:** 2015-05-14

**Authors:** Lily Lessard, Louise Fournier, Josée Gauthier, Diane Morin

**Affiliations:** 1 Department of Nursing Sciences, Université du Québec à Rimouski, Levis and Rimouski, Canada; 2 Faculty of Nursing Sciences, Université Laval, Québec, Canada; 3 Department of Social and Preventive Medicine, Faculty of Medicine, CRCHUM, Université de Montréal, Montreal, Canada; 4 Expert in Organization of Care and Services in Rural and Remote Areas, Health and Social Service System Analysis and Evaluation, Institut national de santé publique du Québec, Rimouski, Canada; 5 Institut universitaire de recherche et de formation en soins, University of Lausanne, Lausanne, Switzerland

**Keywords:** MESH, care pathways, depression, anxiety disorders, continuity of care, Inuit, Community Mental Health Services, Primary Health Care, Quality of Patient Care, Rural Health Services

## Abstract

**Background:**

*Changing Directions, Changing Lives*, the Mental Health Strategy for Canada, prioritizes the development of coordinated continuums of care in mental health that will bridge the gap in services for Inuit populations.

**Objective:**

In order to target ways of improving the services provided in these contexts to individuals in Nunavik with depression or anxiety disorders, this research examines delays and disruptions in the continuum of care and clinical, individual and organizational characteristics possibly associated with their occurrences.

**Design:**

A total of 155 episodes of care involving a common mental disorder (CMD), incident or recurring, were documented using the clinical records of 79 frontline health and social services (FHSSs) users, aged 14 years and older, living in a community in Nunavik. Each episode of care was divided into 7 stages: (a) detection; (b) assessment; (c) intervention; (d) planning the first follow-up visit; (e) implementation of the first follow-up visit; (f) planning a second follow-up visit; (g) implementation of the second follow-up visit. Sequential analysis of these stages established delays for each one and helped identify when breaks occurred in the continuum of care. Logistic and linear regression analysis determined whether clinical, individual or organizational characteristics influenced the breaks and delays.

**Results:**

More than half (62%) the episodes of care were interrupted before the second follow-up. These breaks mostly occurred when planning and completing the first follow-up visit. Episodes of care were more likely to end early when they involved anxiety disorders or symptoms, limited FHSS teams and individuals over 21 years of age. The median delay for the first follow-up visit (30 days) exceeded guideline recommendations significantly (1–2 weeks).

**Conclusion:**

Clinical primary care approaches for CMDs in Nunavik are currently more reactive than preventive. This suggests that recovery services for those affected are suboptimal.

Over the past 20 years, mental health problems and psychological distress have increased among the populations of Inuit Nunangat (homeland of the Canadian Inuit) (1–3). Given the potential impact on those affected, their loved ones and individuals living in northern communities (4,5), it is important to act on multiple determinants such as access to quality local healthcare (1).

Healthcare services in remote regions face several geographical, organizational and cultural constraints that can bring about inconsistencies in mental health clinical approaches (1,2,6–8). Therefore, to bridge critical gaps in the provision of services for Canada's Inuit, national mental health action plans make the development of coordinated continuums of care a priority (1,6). However, implementation of adapted and sustainable solutions must be determined by contextualized information.

This article presents the results of research that documented the continuum of care for individuals with common mental disorders (CMDs) living in northern communities. The disorders referred to are depression and anxiety. Because CMDs are so often underdiagnosed, few reliable data are available on the epidemiology of these disorders in Inuit Nunangat (4,5,9,10). Nevertheless, based on measured levels of psychological distress and the presence of numerous mental health risk factors, it is generally agreed that there is high prevalence (2,3,9,10).

## Current clinical guidelines for CMDs

CMDs are often associated with increased suicide risk and significant functional impairment at professional, academic, social and family levels (11). Due to the high risk of relapse, these disorders are increasingly regarded as chronic conditions. Current general guidelines encourage frontline health and social services (FHSSs) to manage most affected individuals, while reserving specialized services for more complex cases (12,13). For FHSSs to contribute effectively to an individual's recovery on the psychosocial, emotional and community level, the least that is expected is (13–15):

- improved accessibility
- rapid detection and adequate assessment of CMDs
- early intervention to limit functional impairment, that takes into account the individual's condition, treatment and medical history, risk factors and preference
- long-term follow-ups to ensure continuous monitoring of the individual's status, so that treatment can be adjusted and also to encourage adherence to the treatment plan.

## FHSSs in Inuit Nunangat

FHSSs are generally described as the first level of universally accessible services that promote health, prevent diseases and provide diagnostic, curative, rehabilitative, support and palliative services (16). Their design depends on where they are developed (17). In Inuit Nunangat, as in other remote regions, FHSS teams are multidisciplinary and healthcare providers generally play a major role in meeting the population's essential healthcare needs. General practitioners support nurses and social workers locally or remotely. In communities with no permanent doctors, medical visits can be organized and access to specialized mental health resources is usually dependent upon service agreements with health facilities in urban areas in the south.

## Objectives

This research aims to document the continuum of care for individuals with a CMD who consult FHSSs in Nunavik, one of Inuit Nunangat's 4 regions. The authors have addressed the continuum of care in terms of continuity of contact, that is, the maintenance of care over time to create a continuum of services that satisfy the individual's needs (18). Our objectives were to identify: (a) delays and disruptions in the continuum of care; and (b) clinical, individual and organizational factors possibly associated with such occurrences.

### Study setting

Nunavik is a 505,000 km^2^ region located north of the 55th parallel in Quebec province. In 2006, 91% of the region's 10,784 inhabitants self-identified as Inuit (19). The population is spread among 14 communities on the shores of Hudson Bay and Ungava Bay. These communities are fly-in villages. They have no road connection to each other or to Quebec's road network. The main method of transportation is by plane and this is very costly and time-consuming.

**Fig. 1:**
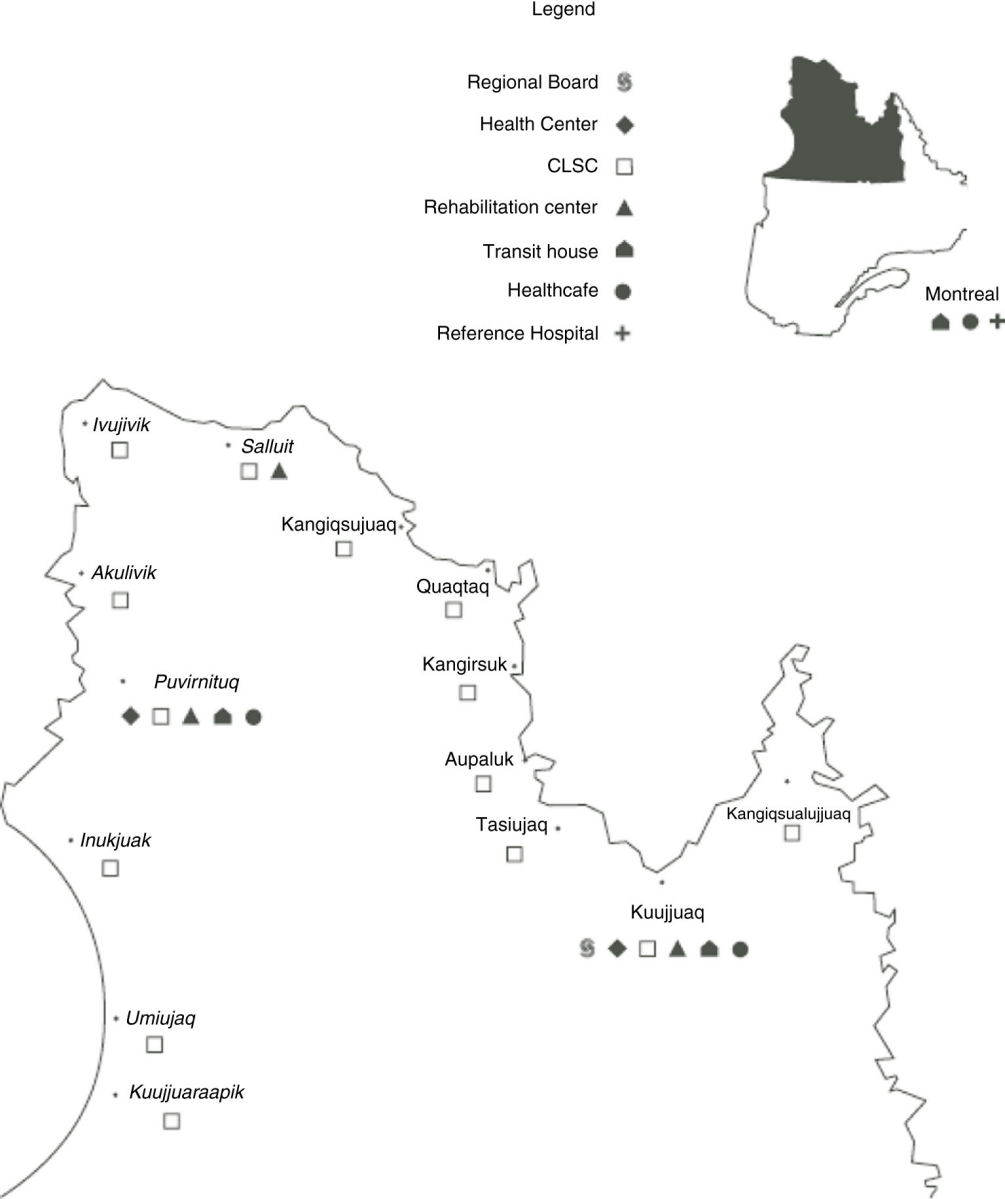
Nunavik health and social services reproduced with permission from Nunavik Regional Board of Health and Social Services.

Nunavik's health infrastructure has been part of Quebec's healthcare system since 1975 (20). The Nunavik Regional Board of Health and Social Services organizes health and social services programs and coordinates budget allocations for 2 sub-regional healthcare centres that offer general hospital services to the populations of Ungava Bay and Hudson Bay. Primary nursing care and social services are available at the local health centre in each community, referred to locally as the nursing station. Local centres are linked administratively to one of the larger healthcare centres, either Tulattavik Health Centre (Ungava Bay) or Inuulitsivik Health Centre (Hudson Bay) (Fig. 1). For mental health, service agreements with specialized facilities in Montreal enable FHSS teams to organize regular visits by psychiatrists and child and adolescent psychiatrists in the region; provide remote support to FHSS teams; and guarantee access to hospital beds. During this study, the region had only one adult psychologist working at the Tulattavik (Ungava Bay) healthcare centre. Since 1999, Nunavik has implemented infrastructure dedicated in whole or in part to individuals with mental health problems (crisis centre, reintegration centre for individuals with severe mental disorders, homes for troubled youth, supervised apartments and drug treatment centres).

## Methods

### Study design

This study is based on a descriptive correlational design where the continuum of care is studied for time-limited episodes. These episodes refer only to the occurrence of a CMD or relapse. Each episode begins at the first sign and symptom of a CMD and ends after the second follow-up visit.

### Population and sample

The study population comprised FHSS users in Nunavik, aged 14 years and older who experienced at least one CMD-associated episode of care (occurrence or relapse) during an observation period.

### Data source

Researchers documented episodes of care extracted from 79 clinical records of FHSS users in Nunavik, who were aged 14 years or older on 1 January 2007 and who had depression or an anxiety disorder. Data were collected in 2009 in 10 Nunavik communities as part of a research program on the quality of mental healthcare (21). The data were recorded in an Access database and described all the consultations of individuals presenting with suspected or confirmed CMDs over a 2-year observation period. This period was established by using the last mental health consultation in 2007 as point of reference and then adding the previous and the following 12 months. The presence of a CMD was confirmed after considering the diagnoses documented during the observation period or, in their absence, by establishing research diagnoses from signs and symptoms presented by the individual during this period. The data collection process, developed tools and data preparation were validated using a rigorous iterative approach. This data collection process is detailed in another article (22).

### Examining episodes of care

A total of 155 episodes of care were reconstructed. Each episode of care was divided into 7 stages: (a) detection; (b) assessment; (c) intervention; (d) planning the first follow-up visit; (e) implementation of the first follow-up visit; (f) planning a second follow-up visit; (g) implementation of the second follow-up visit. The delay to carry out each stage and identify interruptions in the continuum of care was based on the sequential examination of the stages. Table I shows the conditions for success, the delays and the interruptions for each stage.

**Table I:** Conditions for success, delays and interruptions at each stage of an episode of care

Stages	Success	Delay	Interruptions
1. Detection	The healthcare provider briefly assesses the individual's mental state, or refers them to someone else for this purpose, in the presence of signs or symptoms of depression or anxiety disorders (26).	Detection delay=0: Detection occurs after the first visit. Detection delay=1: Detection occurs during a subsequent visit planned by the healthcare provider.	Break=1: The healthcare provider does not make a brief examination of the mental state, or does not refer the individual to obtain one, when there are signs and symptoms of depression or anxiety disorders (26) AND a follow-up visit is not planned.
2. Initial assessment	Following detection, an assessment plan developed by the healthcare provider is completed.	Assessment delay=0: Assessment is carried out in one visit. Assessment delay=1: Assessment is carried out in more than one planned visit.	Break = 1: The assessment plan provided by the healthcare provider is not completed.
3. Initial intervention	At the end of the assessment, treatment^a^ is carried out. OR Treatment is not carried out after the assessment, but a follow-up is planned (vigilant observation).	Intervention delay=0: Treatment starts at the end of the assessment. Intervention delay=1: Treatment does not occur immediately after the end of the assessment. These delays are usually due to referrals to another healthcare provider or service.	Break=0: No treatment is provided, but the individual's condition does not require it or the individual does not want to be treated. Break=1: No treatment is provided and no follow-up is planned even though the individual's condition requires it.
4. Planning the first follow-up	An initial follow-up is planned after the assessment or treatment.	Delay between planning and implementation of the first follow-up (in number of days).	Break=0: No follow-up visit planned, but the individual's condition does not require it or the individual does not want to be monitored. Break=1: First follow-up is not planned even though the individual's condition requires it.
5. Implementation of the first follow-up	The first follow-up is implemented.^b^		Break=0: Planned first follow-up visit is not implemented, but the individual's condition does not require it or the individual does not want to be monitored. Break=1: Planned first follow-up visit is not implemented even though the individual's condition requires it.
6. Planning the second follow-up	A second follow-up is planned after the first follow-up is implemented.	Delay between planning and implementation of the second follow-up (in number of days).	Break=0: No second follow-up visit planned, but the individual's condition does not require it, or the individual does not want to be monitored. Break=1: Second follow-up is not planned even though the individual's condition requires it.
7. Implementation of the second follow-up	The second follow-up is implemented.^b^		Break=0: Planned second follow-up visit not implemented, but the individual's condition does not require it or the individual does not want to be monitored. Break=1: Planned second follow-up visit is not implemented even though the individual's condition requires it.

a The interventions identified were: education, helping relationship, observation, anxiolytics given STAT or PRN, prescription of anxiolytics for the long term, referral to social services or community resources for support, prescription for antidepressants, referral to a psychologist or detoxification services, prescription for antipsychotic drugs to potentiate the effect of an antidepressant and referral to a psychiatrist for treatment.

b Follow-up can be implemented at a scheduled time or at another time.

All interruptions that occurred during detection and assessment were deemed breaks in the continuum of care. For stages related to interventions and the 2 follow-up visits, the interruption was not viewed as a break if the individual did not want to be treated or monitored, or if their condition did not require it. According to CMD guidelines (13,15), the need to treat or monitor an individual is associated with the presence of 1 of 3 conditions:

- a moderate to severe CMD diagnosis
- symptoms of CMD in patients with a history of CMD, an alcohol or drug problem or a chronic or serious physical illness
- symptoms of CMD in patients with significant functional impairment.

When this information was not available, the Global Assessment of Functioning scale (GAF) was used to establish a functioning score that took into account medical history plus the type, duration and intensity of the reported symptoms (11). A score of ≤61 meant that function was deemed altered. These scores were established as necessary following interventions and implementation of the first follow-up.

### Influence of clinical, organizational and individual characteristics

#### Dependent variables

Dependent variables correspond to (a) breaks occurring at each stage in the continuum of care and overall and (b) delays assessed for each stage. Success, interruptions, detection delays, assessment and interventions are dichotomous categorical variables. Delays in conducting first and second follow-up visits are continuous variables calculated in number of days.

#### Independent variables

Independent variables correspond to clinical characteristics of episodes of care and to individual and organizational characteristics that can influence the use and provision of services for individuals presenting with CMDs (13,15). These variables are dichotomous.

Episodes of care were first categorized as depression and anxiety based on diagnosis. Where no diagnosis was available, prevalence of signs and symptoms was used. Episodes with both anxiety and depressive components were classed as depression since guidelines recommend treating depression first (13). Episodes were then categorized as incident or relapse, depending on CMD history. The history could have been documented previously or during the observation period. Finally, diagnoses made during episodes of care were categorized as precise or imprecise. A precise diagnosis was (a) made by a person qualified to assess mental disorders (in Quebec province, a physician or psychologist) and (b) not accompanied by other potential diagnoses. Diagnostic accuracy was determined from the assessment stage.

Individuals’ characteristics included age, sex, ethnicity, presence of chronic or severe physical illness and problems associated with alcohol or substance abuse (13). There were 2 age groups (14–20 years and 21 and over) to ascertain whether there were differences between youth and adult services. Ethnicity was based on whether or not the individual was Inuk, which was determined by surname and medical history (anamnesis). Physical illnesses had to have been present during the observation period and related to certain conditions: diabetes, cardiac or chronic respiratory problems, rheumatoid arthritis, chronic pain, cancer, severe sensory limitations, epilepsy, thyroid imbalances and other conditions causing significant distress. The presence of at least one note during the observation period that reported regular use or abuse of alcohol or illicit drugs established alcohol or drug-related problems (23).

Organizational characteristics refer to the Tulattavik Health Centre (Ungava Bay) and the Inuulitsivik Health Centre (Hudson Bay), as well as the FHSS teams. Teams were classed as limited or expanded according to whether or not a permanent doctor was available on-site.

### Statistical analysis

Descriptive statistics were used to highlight delays, interruptions and breaks at each stage in the continuum of care. The impact of the independent variables on breaks and delays was then examined using comparative analysis. First, bivariate analysis was carried out. Analyses involving clinical characteristics of episodes of care used chi-square tests for the categorical dependent variables and t-tests for continuous dependent variables. Logistic and linear regression models were used for individual and organizational characteristics. Since a single individual could have more than 1 episode of care (24), the models were adjusted using a generalized estimating equation approach (GEE) to take into account data correlations (25). To ensure the validity of multivariate logistic regression models, a rule recommending a minimum of 10 occurrences per independent variable was applied (26). In addition, only variables in the bivariate analysis with p-values less than ≤0.25 were retained in the multivariate regression models. Analyses were performed using SPSS 22 software and results are considered significant at ≤0.05.

### Ethical approvals

This research received administrative approval from the participating healthcare centres as well as ethical certification from the *Agence de la santé et des services sociaux* in Montreal and *Université Laval's* research ethics committee.

## Results

### Sample characteristics

Table II presents the individual and organizational characteristics of the sample (79 individuals). For the episodes, the number of individuals per file varies from 1 to 9 (x=2.1, SD=1.6; md= 2.0) and their characteristics are shown in Table III.

**Table II:** Individual and organizational characteristics of sample (n=79)

*Characteristics*	X±SD (md) (Min–max)
Age	34.9±14.7 (34.0) (14–78)
	N (%)
Age	
14–20 years old	15 (19.0)
≥ 21 years old	64 (81.0)
Sex *(female)*	49 (62.0)
Ethnicity *(Inuit)*	73 (92.4)
Drugs, alcohol *(presence)*	44 (55.7)
Chronic physical illness *(presence)*	40 (50.6)
Team *(expanded)*	55 (69.6)
Health centre	
Inuulitsivik Health Centre (Hudson Bay)	46 (58.2)
Tulattavik Health Centre (Ungava Bay)	33 (41.8)

**Table III:** Clinical characteristics of care episodes (n = 155)

Characteristics	X±SD (md) (Min–max)
Length^a^ (days)	46.7±70.6 (10.0) (1–382)
	N (%)
Symptom predominance	
Anxious	89 (57.4)
Depressive	66 (42.6)
CMD history *(presence)*	87 (56.1)
Diagnosis accuracy *(precise)*	64 (41.3)

a 5 missing data.

Women make up almost two-thirds of the sample. This is consistent with the distribution of CMDs found in the general population (27). More than half the sample population had a serious or chronic physical illness (51%) or alcohol and drug problems (56%). However, this rate is lower than that measured in the population of Nunavik in 2004, where 90% of those 15 and over reported having had at least one alcohol abuse episode and 60% had used illicit drugs during the previous 12 months (23). Nevertheless, this variable was retained for subsequent analysis based on the presumption that recording information on an individual's consumption might be associated with functional impairment for that person. Allocation according to ethnicity, FHSS team and healthcare centre corresponds to what is found in Nunavik's population (19).

The correlation analysis between these variables revealed some significant associations, but only at a mild to moderate intensity (Phi ≤ 0.33). As a result, they were considered sufficiently independent to be retained for the multivariate analysis.

Anxiety episodes outnumber depressive episodes. The majority of anxiety episodes are recurring; that is, there is a history of CMD. Finally, the mental disorder diagnosis was accurate for approximately 40% of episodes. The correlation analysis between the variables Symptom Predominance, Presence of CMD History and Diagnosis Accuracy revealed significant associations of mild to moderate intensity (Phi ≤ 0.26).

### Examining episodes of care

**Fig. 2:**
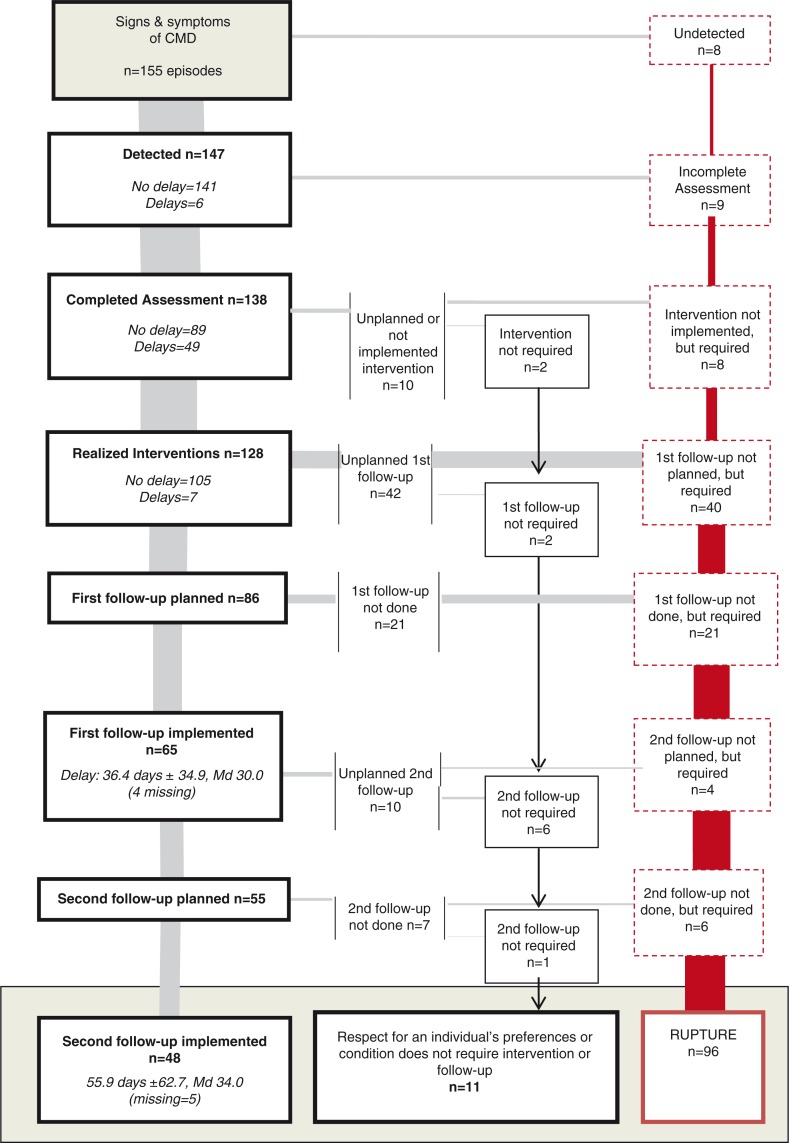
Continuum of care.

Figure 2 presents the success, delays and interruptions for each stage in the episode of care. Of the 155 initial episodes, 48 (31%) were pursued until the second follow-up was conducted and 107 (69%) were interrupted during one of the other previous stages. Of these interruptions, 11 (7%) involved conditions that did not require interventions or follow-ups or were associated with the individual's preferences. The other 96 (62%), showed breaks in the continuum of care that occurred mainly during planning of the first follow-up visit (n = 40; 42%) and at its implementation (n = 21; 22%). For most episodes of care, detection and interventions were carried out without delay. For the assessment stage, more than a third of the episodes required more than 1 visit. The median time for first and second follow-ups was 30.0 days (x=36.4±34.9) and 34.0 (x=55.9 days±62.7), respectively.

### Factors associated with breaks in the continuum of care

Bivariate analyses were carried out between breaks and certain clinical, individual and organizational characteristics. The bivariate analyses are shown in Table IV. Only breaks that occurred in the overall continuum of care as well as stages involving planning and implementation of the first follow-up visit showed a sufficient number of occurrences to perform multivariate analyses. The results of these analyses, expressed as odd ratios (OR), are shown in Tables V,VI and VII. An OR > 1 means an increased risk for a break in the continuum of care while OR < 1 indicates a lower risk.

**Table IV:** Bivariate analyses for interruptions in continuum of care

	Rupture at detection (N=155)	Rupture at assessment (N=147)	Rupture at intervention (N=138)	Rupture at plan.1st follow-up (N=128)	Rupture at real.1st follow-up (N=86)	Rupture at plan.2nd follow-up (N=65)	Rupture at real.2nd follow-up (N=55)	Rupture overall sequence (N=155)
								
Characteristics	8 (5.2%)	9 (6.1%)	8 (5.8%)	40 (31.3%)	21 (24.4%)	4 (6.2%)	6 (10.9%)	96 (61.9%)
Episodes of care^a^								
Predominance								
Anxious (%)	6 (6.7)	2 (2.4)*	8 (9.9)*	34 (46.6)*	17 (43.6)*	2 (9.1)	4 (22.2)§	73 (82.0)*
Depressive (%)	2 (3.0)	7 (10.9)	0 (0)	6 (10.9)	4 (8.5)	2 (4.7)	2 (5.4)	23 (34.8)
Common MH Hist.								
Yes (%)	5 (5.7)	7 (8.5)§	6 (8.0)§	24 (35.3)	16 (29.5)§	3 (9.7)§	5 (17.9)§	63 (72.4)*
No (%)	3 (4.4)	2 (3.1)	2 (3.2)	16 (26.7)	8 (19.0)	1 (2.9)	1 (3.7)	33 (48.5)
Diagnostic								
Precise (%)	–	–	0 (0)*	10 (16.7)*	9 (18.0)§	1 (2.4)§	1 (2.9)*	23 (24.0)*
Imprecise (%)	–	–	8 (10.5)	30 (44.1)	12 (33.3)	3 (12.5)	5 (23.8)	73 (80.2)
Individual and Organizational^b^								
Age group								
14–20 years old (%)	0 (0)	3 (14.3)§	1 (5.6)	3 (17.6)	1 (7.1)	0 (0)	0 (0)	8 (38.1)§
≥ 21 years old (%)	8 (6.0)	6 (4.8)	7 (5.8)	37 (33.3)	20 (27.8)	4 (7.7)	6 (13.3)	88 (65.7)
Sex								
Female (%)	4 (4.2)	4 (4.4)§	6 (6.9)	19 (24.1)§	16 (27.6)	2 (4.8)	2 (22.2)§	53 (55.8)*
Male (%)	4 (6.7)	5 (8.9)	2 (3.9)	21 (42.9)	5 (17.9)	2 (8.7)	4 (5.4)	43 (71.7)
Ethnicity								
Inuit (%)	8 (5.4)	9 (6.5)	8 (6.2)	40 (33.3)	20 (25.6)	4 (6.9)	4 (8.3)§	93 (63.3)
Non-Inuit (%)	0 (0)	0 (0)	0 (0)	0 (0)	1 (12.5)	0 (0)	2 (28.6)	3 (37.5)
Alcohol or drug abuse								
Yes (%)	0 (0)	7 (7.8)	5 (6.0)	26 (33.3)	12 (23.1)	2 (5.0)	5 (15.2)§	57 (63.3)
No (%)	8 (12.3)	2 (3.5)	3 (5.5)	14 (28.0)	9 (26.5)	2 (8.0)	1 (4.5)	39 (60.0)
Physical Illness								
Yes (%)	7 (8.0)§	3 (3.8)§	6 (7.8)§	25 (35.7)	7 (15.9)	2 (5.4)	4 (12.9)	54 (62.1)
No (%)	1 (1.5)	6 (9.0)	2 (3.3)	15 (25.9)	14 (33.3)	2 (7.1)	2 (8.3)	42 (61.8)
FHSS team								
Expanded (%)	8 (7.9)	4 (4.3)§	6 (6.7)	18 (22.2)*	13 (21.3)	2 (4.2)	3 (7.1)§	54 (53.5)*
Limited (%)	0 (0)	5 (9.3)	2 (4.1)	22 (46.8)	8 (32.0)	2 (11.8)	3 (23.1)	42 (77.8)
Health Centres								
Tulattavik (Ungava Bay) (%)	1 (1.6)§	5 (8.2)	2 (3.6)§	18 (34.0)§	12 (35.3)§	2 (9.1)	3 (8.1)§	43 (69.4)§
Inuulitsivik (Hudson Bay) (%)	7 (7.5)	4 (4.7)	6 (7.3)	22 (29.3)	9 (17.3)	2 (4.7)	3 (16.7)	53 (57.0)

a Bivariate analysis with χ^2^

b Bivariate analysis with logistic regression adjusted with GEE approach

* *p*≤0.05

§ 0.05<*p*≤0.25.

**Table V:** Logistic regressions for interruptions to total continuum of care (n=155)

Variables	Regression coefficient	Standard error	Odds ratio (95% CI)	*p*
Episodes of care^a^				
Predominance (anxious)	1.89	0.42	6.63 (2.91–15.11)	0.000
Common MH history (yes)	0.81	0.42	2.24 (0.98–5.14)	0.055
Diagnosis (precise)	−1.89	0.42	0.15 (0.07–0.35)	0.000
Intercept	−0.06	0.38	0.95	0.882
Individual and organizational^b^				
Age (≤20 years old)	−1.06	0.50	0.35 (0.13–0.92)	0.033
Sex (F)	−0.65	0.49	0.52 (0.20–1.35)	0.100
Expanded FHSS team (yes)	−0.86	0.52	0.42 (0.15–1.18)	0.179
Health centre (Inuulitsivik Hudson Bay)	−0.14	0.50	0.87 (0.33–2.30)	0.773
Intercept	0.675	0.64		0.289

a Nagelkerke R^2^=0.451.

b Logistic regression model adjusted with GEE approach.

**Table VI:** Logistic regressions for interruptions when planning a first follow-up (n=128)

Variables	Regression coefficient	Standard error	Odds ratio (95% CI)	*p*
Episodes of care^a^				
Predominance (anxious)	1.77	0.50	5.89 (2.20–15.78)	0.000
Diagnosis (precise)	−1.099	0.45	0.33 (0.14–0.80)	0.014
Intercept	−1.54	0.47		0.001
Individual and organizational^b^				
Sex (F)	−0.73	0.44	0.48 (0.20–1.3)	0.092
Expanded FHSS team (yes)	−1.08	0.48	0.34 (0.13–0.87)	0.024
Health centre (Inuulitsivik Hudson Bay)	0.171	0.49	1.19 (0.45–3.10)	0.728
Intercept	0.18	0.41		0.669

a Nagelkerke R^2^=0.263.

b Logistic regression model adjusted with GEE approach.

**Table VII:** Logistic Regressions for interruptions at the implementation of a first follow-up (n=86)

Variables	Regression coefficient	Standard error	Odds ratio (95% CI)	*p*
Episodes of care^a^				
Predominance (anxious)	2.025	0.63	7.58 (2.22–25.89)	0.001
Diagnosis (precise)	−0.39	0.56	0.68 (0.23–2.04)	0.491
Intercept	−2.12	0.63		0.001

a Nagelkerke R^2^=0.243.

Analyses first performed on clinical characteristics of episodes show that predominantly anxious episodes are 6–7.5 times more likely to result in a premature break in the overall continuum (OR 6.63, p=0.000) than are predominantly depressive episodes, when planning a first follow-up visit (OR=5.89, p=0.000) and at its implementation (OR=7.58, p=0.001). By contrast, Table IV shows that breaks during the assessment stage would mainly affect depressive episodes (χ^2^=4.57, p=0.032). For diagnostic accuracy, episodes associated with a precise diagnosis were less likely to experience a break than those categorized as imprecise in the overall continuum (OR=0.15, p=0.000) and in the planning of a first follow-up visit (OR 0.33, p=0.014). It would not influence the implementation of the first follow-up (OR=0.68, p = 0.491), but bivariate analysis indicated that less breaks occurred at the second follow-up for episodes of care showing a precise diagnosis (χ^2^=5.82, p=0.016). When there was a history of CMD, only one trend was observed for episodes dealing with a CMD relapse that would have shown more breaks in the overall continuum than for episodes considered incidents (OR=2.25, p=0.055).

For individual and organizational characteristics, only age group and team type showed significant differences. Individuals 14–20 years old were more likely to extend across the entire continuum (OR=0.35, p=0.033) compared to the ≥21 age group. For team type, episodes involving the expanded teams had fewer breaks when planning a first follow-up visit when compared to limited’ teams (OR=0.34, p=0.024).

### Factors associated with delays in the continuum of care

Table VIII shows delays based on independent variables. No significant differences were found for delays at the detection and intervention stages. For the assessment stage, a significant difference in diagnostic accuracy (Table IX) was observed. The assessment of episodes with precise diagnoses was conducted over more than 1 visit whereas imprecise diagnosis took place in 1 visit (OR=3.58, p=0.001). *T*-tests showed no significant difference when estimating effects of the clinical characteristics of episodes on delays in implementing the first and second follow-up visits. Estimating the effects of individual and organizational characteristics on delays in conducting the first and second follow-up visits using bivariate linear regression models, necessitated the conducting of a log transformation for time to ensure normal sample distribution. The results involving these stages refer to Exp (β)-1, which represents how much longer the delay was when an individual presented the studied factor. These analyses showed that the non-Inuit had a 203% (exp (β)-1=2.03; p=0.002) longer delay for the first follow-up visit than the Inuit, and that individuals aged 14–20 years had a 55% (exp (β)-1 =0.55; p=0.046) longer delay for the second follow-up visit than those aged 21 and over.

**Table VIII:** Bivariate analysis of delays occurring at each step of the continuum of care

	Delay at detection N=147	Delay at assessment N=138	Delay at intervention N=112	Delay at 1st follow-up N=61	Delay at 2nd follow-up N=43
					
	6 (4.1%)	49 (35.5%)	7 (6.3%)	36.4 days±34.9	55.9 days±62.7
					
Characteristics	N (%)	N (%)	N (%)	x±SD	x±SD
Episodes of care^a^^b^					
Predominance					
Anxious	4 (4.8)	25 (30.9)§	3 (4.7)	32.9±29.6	39±26.0
Depressive	2 (3.1)	24 (42.1)	4 (8.3)	38.2±37.4	62.1±71.4
Common MH history					
Yes	3 (3.7)	25 (33.3)	2 (3.4)§	31.5±37.0	64.0±76.4
Non	3 (4.6)	24 (38.1)	5 (9.4)	41.6±32.4	46.5±41.7
Diagnostic					
Precise	−	32 (51.6)*	3 (5.7)	38.1±38.2	58.7±70.0
Imprecise	−	17 (22.4)	4 (6.8)	33.5±28.8	49.4±42.6
Individual and organizational^c^^d^					
Age group					
14–20 year olds	0 (0)	8 (44.4)	2 (14.3)§	43.6±10.2	69.5±12.3*
≥ 21 year olds	6 (4.8)	41 (34.2)	5 (5.1)	34.7±5.1	52.7±11.3
Sex					
Females	5 (5.5)§	32 (36.8)	4 (6.0)	39.5±6.8	58.1±12.3
Males	1 (1.8)	17 (33.3)	3 (6.7)	31.4±6.8	50.7±14.1
Ethnicity					
Inuit	6 (4.3)	45 (34.6)	7 (6.7)	35.6±5.2*	58.6±10.7
Non-Inuit	0 (0)	4 (50.0)	0 (0)	42.7±5.5	35.4±10.0
Alcohol or drug disorders					
Yes	3 (3.3)	28 (33.7)	4 (5.8)	36.9±6.2	58.5±14.7
No	3 (5.3)	21 (38.2)	3 (7.0)	35.6±6.9	52.2±10.6
Physical illness					
Yes	4 (5.0)	30 (39.0)§	5 (8.1)	30.0±4.7	65.0±16.8
Non	2 (3.0)	19 (31.1)	2 (4.0)	44.5±8.1	45.4±7.1
FHSS team					
Expanded	4 (4.3)	32 (36.0)	4 (5.6)	36.0±5.8	56.3±11.7
Limited	2 (3.7)	17 (34.7)	3 (7.5)	37.6±7.4	54.1±12.2
Health centre					
Tulattavik Ungava Bay	2 (3.3)	19 (33.9)	0 (0)	35.3±9.6	75.9±24.2
Inuulitsivik Hudson Bay	4 (4.7)	30 (36.6)	7 (10.4)	37.0±5.0	46.2±7.7

* *p*≤0.05

§ 0.05≤*p*≤0.25.

a Bivariate analysis with Chi-square for delays at detection, assessment and interventions.

b Bivariate analysis with t-test for delays at implementation of first and second follow-ups.

c Bivariate analysis with logistic regression model adjusted with GEE approach for delays at detection, assessment and interventions.

d Bivariate analysis with logistic regression model adjusted with GEE approach for delays at implementation of first and second follow-ups, using log of duration due to non-normality of residuals.

**Table IX:** Logistic regression analysis of delays occurring at assessment

Variables	Regression coefficient	Standard error	Odds ratio (95% CI)	*p*
Episodes of care				
Predominance (anxious)	−0.112	0.39	0.89 (0.41–1.93)	0.776
Diagnosis (precise)	1.28	0.39	3.58 (1.67–7.71)	0.001
Intercept	−1.16	0.39	0.312	0.003
Model=63.0				

Nagelkerke R^2^=0.123.

## Discussion

The study's main objective was to document the continuum of care for episodes of care involving individuals with CMD in Nunavik. Most importantly, the results show that a little less than a third of episodes of care get as far as the second follow-up visit. They also indicate that most interruptions in episodes of care involve breaks in the continuum of care. These interruptions generally occur during the planning and implementation of the first follow-up visit, which makes these the most critical stages in episodes of care. Examination of delays showed that for a third of the episodes of care, the assessment necessitated more than 1 visit to be complete. Even so, these delays were associated with more precise diagnoses. The delays measured at the first follow-up (35.6 days±34.14, md=30 days) and at the second (55.9 days±62.7, md=34.0 days) easily surpassed those suggested in the guidelines. The guidelines recommend that the individual be seen again 7–14 days after the initial assessment and that closer monitoring be implemented after that (13,15).

The second objective was to examine whether clinical, individual and organizational characteristics were associated with breaks and delays at various stages in the continuum of care. When breaks occurred, clinical characteristics of episodes of care (prevalence of symptoms and diagnosis accuracy) were most often the cause. For individual and organizational variables, only age (for the continuum as a whole) and composition of the FHSS team (for planning of the first follow-up) showed statistically significant associations.

Not planning a first follow-up visit could have led individuals to think they should return only if symptoms reappeared or worsened. This practice may point towards the adoption of an acute illness management method for CMDs. Yet, current service guidelines for individuals with CMDs advocate follow-up mechanisms based on management models for chronic diseases (13,15,28,29). These responsive clinical practices in FHSS teams were also found in other studies conducted in Nunavik (7) and other remote regions (30,31). The studies show that these FHSS teams are generally ready to respond to crisis situations, but not as ready when it comes to prevention of mental disorders.

The behaviour of healthcare providers and individuals may be the cause of delays and breaks in the implementation of a first follow-up (32). These delays and breaks can be explained by failures in the healthcare system and show, for example, the poor integration of mental health services between health sectors and social services that can be encountered in the context of Aboriginal peoples and remote regions (7,30). Delays and breaks can also be associated with the lack of commitment to treatment plans (31,33) possibly due to how the Inuit of Nunavik perceive depression. Inuit regard depression as a mental health problem that, when it has no hallucinations, does not require medical or professional help and they view anxiety as a transient situation associated with concerns related to difficult life experiences (3). Coincidentally, Kirmayer and Paul (4) reported that in cases of psychological distress, most Nunavimmiut (persons from Nunavik) consult their loved ones rather than FHSS providers (4).

The rate of breaks for depressive-type episodes in Nunavik FHSSs (34.8%) is similar to those documented for depression in the general population (14), but not so for anxiety-type episodes, most of which (82%) end before the second follow-up visit. This could be because healthcare providers tend to get distracted by somatic symptoms, many of which appear to be cardiac related (31,34). Eliminating physical, drug or substance abuse-related causes of somatic symptoms of anxiety disorders and depression is a normal part of a diagnostic approach (11,13). However, our results suggest that even after attributing observed symptoms to anxiety, FHSS care for individuals in Nunavik remains rudimentary. Finally, the fact that, compared to the older age group, episodes among the 14–20 age group persist after the second follow-up, indicates the availability of more sustained approaches for young people. It would be interesting to document these approaches to learn from them and improve the services offered to adults.

### Courses of action

While psychotherapy is often the treatment of choice for individuals with an anxiety disorder (13), this type of treatment is virtually unavailable in Nunavik. Also, results suggest that there are few compensation mechanisms implemented in the region to address the shortage of competent psychotherapists. In similar contexts, initiatives to improve FHSS providers’ cognitive behavioural therapy skills have led to positive experiences when faced with a lack of specialized mental health resources (35,36). Telepsychology, educational interventions and self-care tools adapted for the Inuit are other ways to increase access to quality approaches while promoting empowerment for individuals with anxiety disorders or depression, (31). Results show that more breaks at the planning stage of the first follow-up were documented for the limited FHSS teams. Furthermore, the presence of a precise diagnosis, which suggests the assessment involved a physician or psychologist, meant less premature breaks in the continuum of care. Similar to other studies conducted in remote areas (37), these results show that nurses and social workers should be targeted first in the effort to improve care for individuals with CMDs. Studies have shown that giving nurses a greater role in the treatment and monitoring of individuals with depression significantly increases FHSS quality (38,39).

### Strengths and limits

An important strength of the present study is to identify when delays and breaks occur in mental health care trajectories. This information allows targeting more precisely which clinical aspects (e.g. implementation of a first follow-up) have to be improved in priority to influence the whole continuum of care.

In the present study, success, delays and breaks at each stage in the continuum of care are based solely on FHSS activities and not their nature, quality or individual outcomes. Therefore, further work is needed in Inuit Nunangat to document them. It will lead to a better understanding of how FHSSs contribute to the well-being of individuals with CMDs.

In addition, the continuum of care study was conducted using relatively short episodes that reflect only the early stages of the individual's true care trajectories. Even so, there was sufficient time to expose several gaps and to identify courses of action to improve FHSSs in this region.

Broadening selection criteria beyond the medical diagnosis of depressive and anxiety disorders takes into account the underdiagnosis of these conditions in Inuit Nunangat (4,5). This approach fosters awareness and so can include the disease's prodromal stages in the continuum of care study. This choice is made at the expense of specificity and forces us to proceed with caution when considering the results. Moreover, as most other studies are based on medical diagnoses, there is less possibility for comparison.

The use of clinical records as data sources preserves anonymity and reduces desirability bias while circumventing issues related to the recruitment of participants with mental disorders in such small communities. The quality of a retrospective records review depends largely on the quality of notes within the records. Unfortunately, researchers have little control over this aspect. Consequently, the lack of information in certain records could cause breaks in the continuum of care or skew the effect of certain independent variables, such as alcohol and drug consumption, which appear to be underdocumented in clinical records.

## Conclusion

The study's results support the recent claim of Inuit Tapiriit Kanatami (ITK), an organization representing the Inuit of Canada, which stated that mental health provisions are a patchwork of services, interventions and support measures (1). Poor management leads to chronicity of conditions, reduced quality of life, adoption of harmful behaviours, an increased suicide risk and a worsening of functional limitations (8).

Quality mental health services rely on clinical approaches sustained over time, between healthcare providers and service levels (12,13,15). Also, FHSSs must have a greater impact on recovery. To achieve this, it is important to guide the FHSS teams and those service users with CMD towards chronic disease management methods.

The uniqueness of this study rests upon new data obtained on the quality of FHSSs offered to individuals with CMDs in Inuit Nunangat. It provides a method that can also be reproduced in similar isolated settings, where human and financial resources are limited. It helps us better target courses of action and improve them. Furthermore, observing continuums of care over time could be used to monitor changes made to the healthcare services.
